# Coagulopathy as a Complication of Cardiopulmonary Arrest Following Balloon Valvuloplasty in a Dog

**DOI:** 10.1002/vms3.70445

**Published:** 2025-06-02

**Authors:** Marissa A. Turner, Theresa C. Hallowell

**Affiliations:** ^1^ Emergency and Critical Care Department Ocean State Veterinary Specialists East Greenwich Rhode Island USA

**Keywords:** canine, cardiology, cardiopulmonary resuscitation, pulmonary valve stenosis

## Abstract

**Objective:**

To describe successful management of a coagulopathy secondary to cardiopulmonary arrest (CPA) associated with pulmonary balloon valvuloplasty.

**Case Summary:**

An 8‐month‐old male intact Chihuahua mix presented for a pulmonary balloon valvuloplasty. Echocardiogram showed severe valvular pulmonic stenosis with a pulmonic valve maximum pressure gradient (PV max ΔP) of 108.2 mmHg (PV max ΔP >20 mmHg is considered abnormal). Near the end of the procedure, after the balloon deflation, the patient experienced prolonged periods of sinus arrest with associated ventricular escape rhythm. This was followed by complete sinus arrest and asystole. Cardiopulmonary resuscitation (CPR) was initiated. After four CPR cycles, return of spontaneous circulation was achieved. The patient continued to have hypotension despite fluid resuscitation, requiring vasopressor therapy. Hemorrhagic abdominal effusion was identified. Coagulopathy was confirmed via prolonged blood clotting times and thrombocytopenia. Treatment was comprehensive, including transfusions of whole blood, packed red blood cells and fresh frozen plasma. During 4 days of postoperative hospitalisation, the patient showed improvement and was discharged. Upon rechecking 13 and 227 days after discharge, a repeated echocardiogram showed a >50% improvement of the PV max ΔP measured at 40 and 38.5 mmHg, respectively.

**New or Unique Information Provided:**

This is the first published veterinary case report describing the recognition and successful treatment of a consumptive coagulopathy and presumptive disseminated intravascular coagulation (DIC) following CPA during a balloon valvuloplasty procedure.

## Graphical abstract

An 8‐month‐old dog developed a consumptive coagulopathy and presumptive DIC following cardiac arrest during a balloon valvuloplasty procedure. Successful management required massive transfusion therapy and critical care.

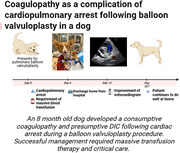

**Abbreviations**
CPA: cardiopulmonary arrest
CPR: cardiopulmonary resuscitation
DIC: disseminated intravascular coagulation
PV: pulmonic valve

## 1 Introduction

Pulmonic stenosis is a commonly observed cardiac defect in dogs. Balloon catheterization is the treatment of choice for relieving clinical signs and prolonging survival associated with the condition (Johnson et al. 2004). Anaesthetic risks have been reported, including hypotension, bradycardia, oxygen desaturation and cardiac arrest. In a retrospective study with 39 dogs undergoing balloon valvuloplasty, only one dog (2.6%) suffered from cardiac arrest due to severe hypotension (Ramos et al. 2014). Reported rates of death in other studies vary from 6.6% to 7.5% due to arrhythmias and hypotension, but all studies have a low number of cases reported (Johnson et al. 2004, Bussadori et al. 2001). Close monitoring and immediate recognition and treatment of complications are required (Ramos et al. 2014). Balloon valvuloplasty can be effective immediately and the effects are long term (Bussadori et al. 2001), helping to relieve clinical symptoms and extend survival times in dogs (Johnson et al. 2004).

Disseminated intravascular coagulation (DIC) is an acquired and life‐threatening syndrome characterised by disseminated and often uncontrolled activation of coagulation. It develops in response to widespread activation of clotting pathways, typically initiated by exposure of tissue factor into the circulation. This is accompanied by enhanced platelet–vessel wall interactions, impaired function of endogenous anticoagulant systems (e.g., antithrombin, protein C) and suppressed fibrinolysis (Papageorgiou et al. 2018). These changes result in the formation of microthrombi throughout the vasculature, leading to organ dysfunction and depletion of platelets and coagulation factors, which paradoxically increases the risk of haemorrhage. The diagnosis of DIC in veterinary patients is based on a combination of clinical signs and laboratory findings. Traditional diagnostic criteria include having at least three of the following abnormalities: prolonged prothrombin time (PT), prolonged activated partial thromboplastin time (aPTT), thrombocytopenia, elevated fibrin degradation products (e.g., D‐dimers), decreased antithrombin activity and evidence of microvascular thrombosis or spontaneous bleeding. Efforts to standardise the diagnosis of DIC have been undertaken by the International Society on Thrombosis and Haemostasis (ISTH). Scoring systems for both overt and non‐overt DIC have been developed and are recommended for clinical use (Taylor et al. 2001). In veterinary medicine, only two studies to date have evaluated DIC scoring systems in dogs, and both identified a significant association between a diagnosis of DIC and increased mortality. However, diagnostic criteria remain variable between studies (Goggs et al. 2018, Wiinberg et al. 2010).

This case report describes the management of presumptive DIC in a patient secondary to cardiopulmonary arrest (CPA) after a balloon valvuloplasty. While coagulopathy and systemic inflammatory responses after CPA are recognised in critical care, the marked hemorrhagic complications occurring in the immediate post‐procedural period highlight a rare and severe manifestation not previously documented. Recognising DIC as a complication in patients undergoing balloon valvuloplasty or experiencing CPA during such procedures, both in human and veterinary medicine, is critical.

## 2 Case Report

An 8‐month‐old male intact Chihuahua mix presented through the cardiology department for a pulmonary balloon valvuloplasty. He initially presented to the cardiology department 64 days prior to surgical intervention for evaluation of an asymptomatic heart murmur. A grade‐V/VI holosystolic murmur was present. Abnormal echocardiogram findings included a dilated right atrium and a dilated right ventricle with paradoxical systolic interventricular septal motion and diastolic flattening. The right ventricular free wall was diffusely thickened. The pulmonic cusps were irregular and redundant. The right ventricular outflow tract gradient was measured at 4.62 m/s (range 1.0–1.8 m/s) (Boon 2011). The pulmonic valve maximum pressure gradient (PV max ΔP) was measured at 85.5 mmHg. Valvular pulmonic stenosis type A was diagnosed. The patient was started on atenolol^a^ (2 mg/kg, PO, q 24 h) for 1 week, and the dose was increased (2 mg/kg, PO, q 12 h) thereafter. A balloon valvuloplasty was scheduled.

**FIGURE 1:**
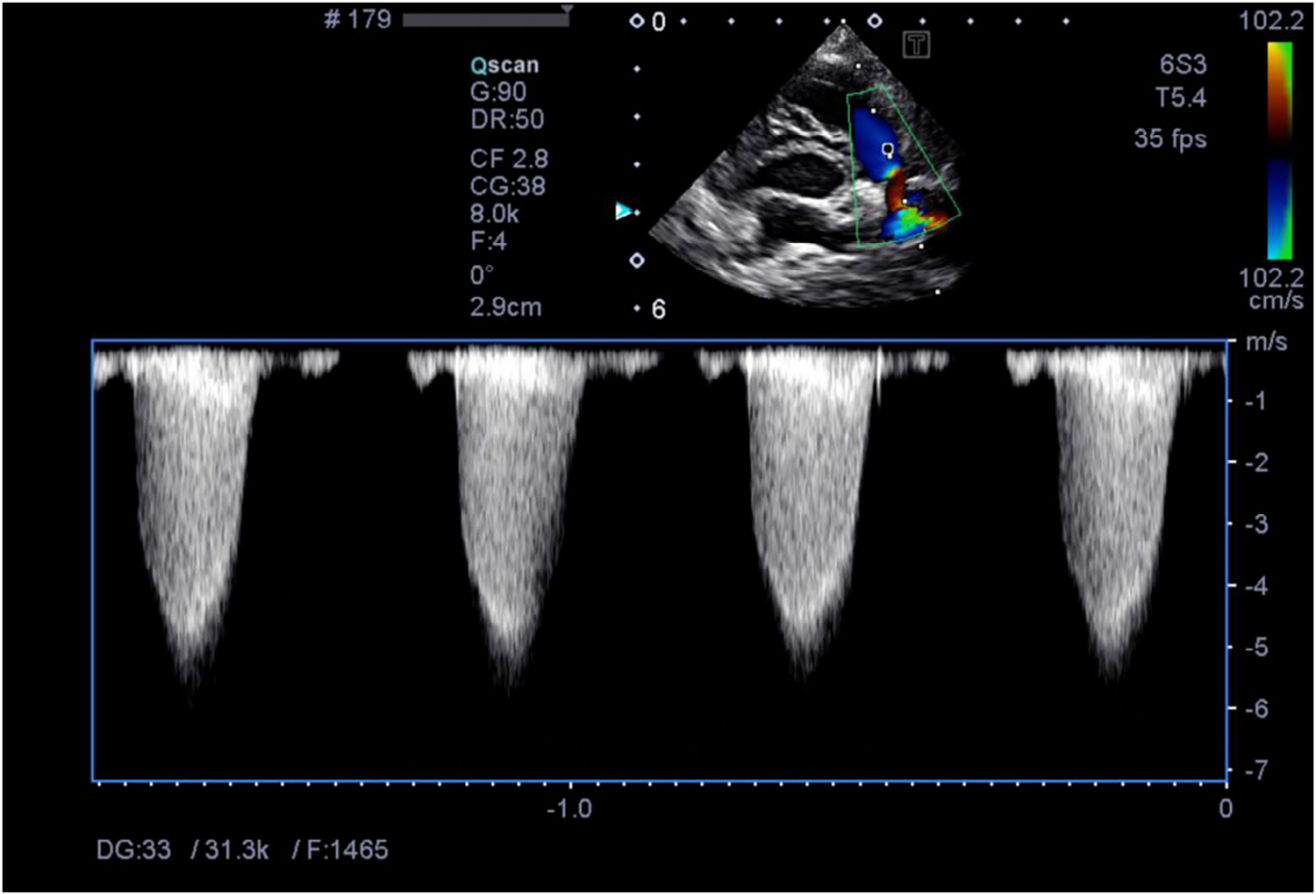
Echocardiographic image prior to balloon valvuloplasty showing colour Doppler imaging of turbulent flow through the pulmonic valve and pulmonic valve prolapse. The spectral Doppler waveform shows a peak velocity exceeding normal physiological limits measured at 4.62 m/s (range 1.0–1.8 m/s) (Boon 2011).

**FIGURE 2:**
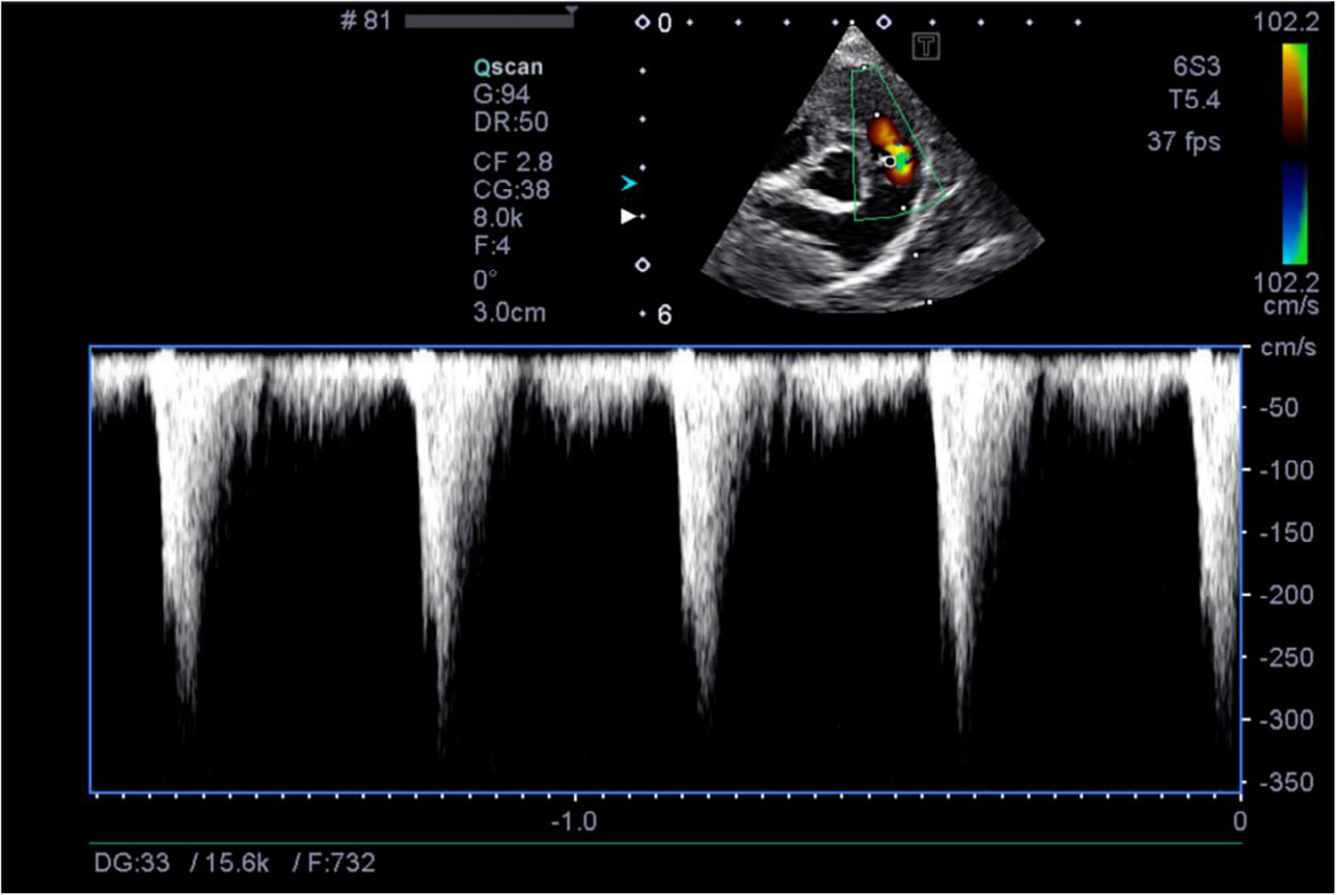
Echocardiographic image post‐balloon valvuloplasty. The colour Doppler image shows an improvement in turbulent flow through the pulmonic valve. The spectral Doppler waveform shows an improved right ventricular outflow tract gradient (cm/s).

The patient presented for his balloon valvuloplasty procedure. His physical exam was unremarkable aside from a static heart murmur. A limited echocardiogram was performed which showed a right ventricular outflow tract gradient of 5.2 m/s and PV max ΔP of 108.2 mmHg. Pre‐op venous blood gas^b^ and electrolyte analysis showed a hypocapnia at 29.8 mmHg (range 30–47 mmHg) and a low base excess of −6.5 mmol/L (range −5 to 5 mmol/L). The packed cell volume and total solids were 53% and 6.8 g/dL, respectively. The patient had an intravenous catheter placed. He was pre‐oxygenated for 10 min prior to anaesthesia induction. Procainamide^c^ (10 mg/kg, IM) was given just prior to pre‐medication along with maropitant^d^ (1 mg/kg, IV). Pre‐medication was provided with butorphanol^e^ (0.19 mg/kg, IV) and diazepam^f^ (0.21 mg/kg, IV). General anaesthesia was induced with Propofol^g^ IV to effect (5.5 mg/kg, IV, required to facilitate intubation). The patient was intubated and maintained on 100% oxygen with sevoflurane gas^h^ (between 1% and 2%) inhalant. A lidocaine^i^ CRI (50 µg/kg/min, IV) was started. During the procedure, ketamine^j^ boluses (0.5 mg/kg, IV) were given twice due to a light plane of anaesthesia. Perioperative antibiotic coverage was provided with cefazolin^k^ (22 mg/kg, IV). A pulmonary balloon valvuloplasty was performed. After the balloon valvuloplasty catheter was deflated and withdrawn from the pulmonary valve into the right ventricle, the patient experienced prolonged periods of sinus arrest, followed by suspected ventricular escape rhythm and occasional ventricular couplets. Complete asystole followed. The procedure was aborted, and chest compressions were initiated using a one‐handed cardiac pump technique due to the small size of the patient. Reversal agents including flumazenil^l^ (0.01 mg/kg, IV) and naloxone^m^ (0.01 mg/kg, IV) were administered, followed by epinephrine^n^ (0.01 mg/kg, IV) and atropine^o^ (0.02 mg/kg, IV). After four rounds of chest compressions, cardiac contractions were noted on fluoroscopy; however, the patient remained bradycardic. A second atropine o dose was given that improved the heart rate from 50–60 to 100–119 beats per minute. Due to hypotension, the patient received a 10‐mL/kg LRS^p^ bolus. Spontaneous breathing returned 15 min after return of spontaneous circulation. Extubation occurred following another 25 min. A blood gas analysis^b^ showed lactic acidosis, with a pH of 7.15 (range 7.360–7.460) and lactate level of 6.76 mmol/L (range 0.6–3 mmol/L). Upon return to the ICU, the patient continued to have hypotension with a systolic pressure of 69 mmHg. He received a 10‐mL/kg LRS^p^ bolus after which he became unresponsive and hypothermic at 95°F. A dopamine^q^ CRI was initiated and titrated up to 5 µg/kg/min to maintain a systolic pressure above 100 mmHg. Point‐of‐care ultrasound showed abdominal effusion. Due to initial concern for right‐sided congestive heart failure, a repeated echocardiogram was performed. The heart appeared volume‐depleted with suspected left‐ and right‐sided pseudohypertrophy. The PV cusps appeared to have improved excursions. Trans‐pulmonic velocity was significantly reduced post‐operatively with a PV max ΔP of 32.5 mmHg. Echocardiographic images obtained pre‐ and post‐balloon valvuloplasty demonstrated improved pulmonic flow gradients (Figures 1 and 2).

The patient's mucous membrane colour became pale white, and an ultrasound‐guided abdominocentesis was performed which yielded hemorrhagic fluid with a PCV of 41% and a TS of 5.0 g/dL. A peripheral PCV was 35% with a TS of 4.6 g/dL. PT was out of range at >100 s (range 11–17 s), and partial thromboplastin time (PTT)^r^ was >300 s (range 72–102 s). A complete blood count^s^ was performed which showed thrombocytopenia of 65,000 × 10^3^/µL (range 117–140 × 10^3^/µL); this was confirmed with a manual platelet count. Consumptive coagulopathy was suspected.

The patient was blood‐typed^t^ DEA 1.1 positive and received 8.3 mL/kg packed red blood cells^u^ over 15 min and another 8.3 mL/kg over 2 h. He also received 22.2 mL/kg fresh frozen plasma^v^ over 2 h. Tranexamic acid^w^ (10 mg/kg, IV) was bolused over 2 min, followed by a 10 mg/kg/h IV CRI for 3 h to support tertiary haemostasis. Vitamin K^x^ (0.55 mg/kg, SQ, q 12 h) and desmopressin^y^ (0.0003 mg/kg, IV) were administered to increase the activation and release of clotting factors into circulation. Blood pressure improved to 104 mmHg systolic, and the patient's mentation improved. During recovery, the patient was observed profusely haemorrhaging from his jugular venipuncture site that required the placement of haemostatic gel foam and a pressure bandage. The patient began having haematochezia. A recheck blood gas assessment showed persistent hyperlactatemia (9.49 mmol/L) and BUN of 32 mg/dL (range 7–26 mg/dL). The patient again became mentally dull, hypothermic and hypotensive. The patient received an additional 8.3 mg/kg of packed red blood cells^u^ over 30 min, followed by 8.3 mg/kg of packed red blood cells^u^ over 2 h. Simultaneously, the patient also received 11 mL/kg fresh frozen plasma^v^ over the same time frame of 2 h. A vasopressin^z^ CRI at 0.015 units/kg/h was added to the therapy along with hydrocortisone^aa^ (2 mg/kg, IV, q 6 h). Due to continued haematochezia and the potential for bacterial translocation, antibiotics were started (enrofloxacin^ab^ 6.6 mg/kg, IV, q 24 h; metronidazole^ac^ 10.1 mg/kg, PO, q 12 h). Additionally, pantoprazole^ad^ (0.9 mg/kg, IV, q 12 h) was administered in anticipation of ischemic gastropathy secondary to systemic shock and coagulopathy. The lidocaine CRI was discontinued. Fentanyl^ae^ at 3 µg/kg/h was started for post‐operative analgesia. Overnight, the patient required further packed red blood cells (10 mL/kg), a whole blood transfusion (5 mL/kg bolus, then a total of 12 mL/kg) and plasma (19 mL/kg) due to continued tachycardia (170 beats per minute) and hypotension (69 mmHg). Recheck PT and PTT were 17 and 105 s, respectively, and by the morning after his procedure, they were within normal limits (15 and 99 s, respectively). After coagulation parameters normalised, a nasogastric tube was placed to provide enteral nutrition. On Day 2 of hospitalisation, a complete blood count^s^ and chemistry panel^af^ showed anaemia, with red blood cells being 4.49 M/µL (5.39–8.7 M/µL), haematocrit 32.6% (38.3%–56.5%) and haemoglobin 11.2 g/dL (13.4–20.7 g/dL). Platelets were estimated to be decreased at 50,000/µL. Elevated liver values were noted; ALT 2457 U/L (18–121 U/L) and ALP 225 U/L (5–160 U/L). The patient was weaned off vasopressors by Day 3 of hospitalisation. On Day 4, the patient was bright alert and responsive, eating and was discharged home due to financial constraints. Follow‐up phone calls were conducted over the following week to ensure the patient was progressing as expected.

**FIGURE 3:**
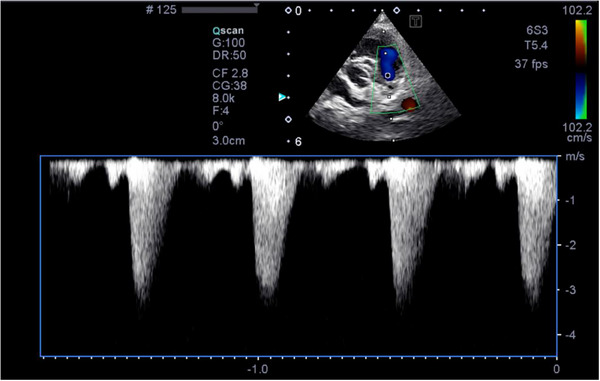
Echocardiographic image 227 days post‐balloon valvuloplasty. The colour Doppler image shows continued improvement of turbulent flow through the pulmonic valve. The spectral Doppler waveform shows an improved right ventricular outflow tract gradient (3.1 m/s).

A recheck examination 13 days after discharge showed >50% improvement of the PV max Δ. The owner reported that he was clinically doing well. A complete blood count^s^ and chemistry panel^ff^ were performed which showed all parameters returned to normal aside from an elevated ALT of 178 U/L (10–125 U/L). Atenolol was restarted. A recheck 227 days after discharge showed continued improvements on repeated echocardiography (Figure 3). The owner still reported that he was doing well clinically.

## 3 Discussion

This case report describes the treatment of a post‐cardiac arrest patient with the development of a severe coagulopathy, presumed to be associated with DIC, following a balloon valvuloplasty procedure.

Many pathological conditions can activate the coagulation cascade, including hypoxia and cardiopulmonary resuscitation (CPR). In veterinary medicine, DIC has been reported as a secondary complication of various disease processes, such as neoplasia (Granger et al. 2024), heartworm adulticide therapy (Philp et al. 2023) and systemic infections (Honse et al. 2013). Experimental studies in animal models have also demonstrated coagulopathy following successful CPR efforts after experimentally induced ventricular fibrillation (White et al. 2011). Despite these findings, there are currently no published clinical reports in veterinary literature describing DIC as a complication specifically following CPR or after balloon valvuloplasty procedures. A retrospective study evaluating complications in canine interventional cardiology procedures, including balloon valvuloplasty, did not identify DIC as a reported outcome (Leblanc et al. 2019).

A review of DIC in human medicine showed that patients suffering from cardiac arrest and resuscitation may have several mechanisms and triggers of DIC, including inflammatory cytokine release, platelet activation and exhaustion, systemic activation of the coagulation system, impairment of anticoagulation pathways, increased level of fibrinolysis followed by inhibition of fibrinolysis, neutrophil activation and endothelial injury (Gando and Wada 2019, Duan et al. 2024). Post‐resuscitation hypoxia has been described, and prolonged resuscitation efforts worsen the degree of coagulopathy, increasing mortality risk (Papageorgiou et al. 2018, Gando and Wada 2019, Duan et al. 2024).

A case report in human medicine described acute DIC after percutaneous balloon aortic valvuloplasty in a patient with liver cirrhosis (Unger et al. 1989). A study discussing long‐term results of aortic balloon valvuloplasty in humans described 13 patients (4.8%) with procedure‐related mortality. One of these patients suffered from DIC (Reich 2004). In human medicine, patients who undergo balloon aortic valvuloplasty have a procedural mortality of 1.4%, with in‐hospital mortality being 8.5%. Blood transfusions were required in 17.5% of cases. Coagulopathy as a complication in this study was reported at 0.09% (Alkhouli et al. 2017). There is also a case report describing catheter ablation, a widely performed minimally invasive procedure for atrial fibrillation in humans, causing DIC (Park et al. 2005).

Coagulation abnormalities have been documented in human paediatric patients with uncorrected congenital heart defects, further underscoring the link between cardiovascular pathology and systemic haemostatic imbalance. A study at a teaching hospital showed that children with uncorrected congenital heart defects frequently exhibit derangements in coagulation profiles, including prolonged PTs, prolonged PTT, thrombocytopenia and elevated D‐dimer levels, even in the absence of overt bleeding. The reported prevalence is 31.7% compared to 7.2% in the healthy cohort. These findings reflect a state of chronic coagulation activation and endothelial dysfunction likely driven by hypoxia, altered haemodynamics and systemic inflammation (Majiyagbe et al. 2022). There is no similar coagulation study performed in veterinary patients with congenital heart defects. In this case, coagulation parameters were not evaluated pre‐operatively, which could be viewed as a limitation; however, there is a lack of evidence in veterinary medicine to support pre‐emptive coagulation screening of patients prior to interventional cardiology procedures.

The pathogenesis of coagulopathy in this case can also be understood through the lens of Virchow's triad, which outlines the three primary contributors to thrombus formation: endothelial injury, altered blood flow and hypercoagulability (Wolberg et al. 2012). All three components were likely at play with this patient. The valvuloplasty procedure and resuscitation efforts likely caused mechanical trauma and endothelial disruption. Altered blood flow resulting from hemodynamic instability and hypotension may have contributed to stasis and impaired tissue perfusion. Lastly, the patient's hypercoagulable state may have been driven by systemic inflammation, surgical stress and tissue hypoxia, all of which are known to activate procoagulant pathways, inhibit fibrinolysis and shift the haemostatic balance towards thrombosis (Wolberg et al. 2012).

DIC is a condition of prothrombosis from systemic coagulation activation due to an underlying disease (Levi and Sivapalaratnam 2018). The diagnosis of DIC is based on clinical suspicion and abnormalities of blood coagulation testing, including PT/PTT, platelet count and fibrinogen levels. There is no gold standard diagnostic test for DIC; instead, diagnosis is based on diagnostic scoring systems (Wiinberg et al. 2010). In veterinary medicine, the consensus is currently lacking. Wiinberg et al. (2010) describe a scoring system based on PTT, PT, D‐dimer and fibrinogen that has a diagnostic sensitivity of 90.9% and specificity of 90.0% based on expert opinion. In a more recent study, using a cutoff of 3 or more abnormal values (PT, aPTT, D‐dimer, decreased AT, fibrinogen and platelet count) was 72.7% sensitive and 80.9% specific for mortality (Goggs et al. 2018). In our case study, D‐dimers, fibrinogen and AT were not available for review due to the urgency of the clinical situation and financial considerations. These values could have provided additional confirmation of DIC in this patient and represent a limitation of this case report. The presumption of DIC was made due to confirmed coagulopathy using coagulation testing (PT/PTT) and platelet count, and its resolution with treatment.

Treatment of DIC includes treatment of the underlying disorder driving the patient's DIC (Levi et al. 2009) and supportive treatments including judicious haemostatic support with blood component therapy and intensive critical care therapies (Bick 2003). DIC is associated with low survival probability and a high rate of in‐hospital mortality (Gando and Wada 2019). In veterinary patients, the mortality rate of dogs with DIC using a scoring system is 62.5% (Goggs et al. 2018). Haemostatic support through blood component therapy played a critical role in this patient's survival. This patient met the criteria for a massive transfusion, which is defined as replenishment of a patient's blood volume over 24 h or greater than or equal to 50% of the estimated blood volume in 3 h. The entire blood volume of a dog is defined as 80 mL/kg of body weight (Tucker et al. 2022). This patient exceeded this at 107 mL/kg of blood products within a 24‐h period. At the 3‐h cutoff, the patient received 38.8 mL/kg of blood products, which is just under 50% of the estimated blood volume. While essential for correcting hemorrhagic shock and coagulopathy, massive transfusions can contribute to or exacerbate DIC. The pathophysiology is complex and, in trauma patients, is thought to be a combination of dilution of clotting factors, platelet depletion and disruption of fibrinolytic balance (Hardy et al. 2006). In this case report, it is possible that the requirement for a massive transfusion may have exacerbated DIC; however, the diagnosis of a consumptive coagulopathy—including a decrease in PCV, out‐of‐range PT and PTT, thrombocytopenia and a hemoabdomen diagnosis—was made prior to haemostatic support.

Limitations to this case study include the presumptive diagnosis of DIC, lack of additional coagulation testing and lack of electrocardiographic tracings. The presumptive diagnosis of DIC was made, as tests such as D‐dimer and fibrinogen were not obtained. In‐house coagulation testing introduces significant variability and limits the ability to definitively diagnose or exclude consumptive coagulopathy as a cause of haemorrhage. Issues such as collection technique, sample handling and equipment malfunction contribute to this variability. While point‐of‐care devices such as the PT IDEXX Coag Dx are generally reliable for detecting coagulopathies, as demonstrated by Yang et al. (2018), these tests still carry inherent limitations. This necessitates a broader diagnostic approach, considering other potential causes of hemoperitoneum, including liver fractures/lacerations secondary to CPR, which has been reported in the veterinary literature to occur in 8.9% of cases (Quesada et al. 2021).

In this case, it is plausible that liver fracture or laceration occurred as a result of compressions during resuscitation rather than being a secondary manifestation of consumptive coagulopathy. Evidence from human and veterinary literature underscores the anatomical susceptibility of the liver and the plausibility of direct trauma leading to laceration (Padmanabhan et al. 2024). The patient was very small in size, and as such, compressions were made directly over the heart (one‐handed cardiac pump technique) rather than the entire thoracic cage. This should result in less direct liver injury, but does not fully eliminate the risk (Kapłon‐Cieślicka et al. 2013).

Lastly, electrocardiogram tracings from the peri‐arrest period were not retained and are therefore unavailable for inclusion in this report. Interpretation of the cardiac rhythm was based on clinical monitoring and fluoroscopic findings. While rhythm disturbances were described in this report, the absence of electrocardiogram tracings precludes independent rhythm interpretation, which would have further strengthened the educational value of the report.

In conclusion, this report provides the first documentation of severe coagulopathy (presumptive DIC) following resuscitation from post‐valvuloplasty cardiac arrest. We acknowledge that as a single‐case report, generalisability is inherently limited, and conclusions should be interpreted with appropriate caution. However, coagulopathy is a recognised complication of congenital cardiac defects, valvuloplasty procedure and CPA in human patients. While coagulopathy has not previously been reported in these conditions in veterinary patients, it is reasonable to suggest that the mechanisms for coagulopathy would be similar. Further study of coagulation parameters in patients with congenital heart defects, particularly during the peri‐procedural period in dogs undergoing valvuloplasty, is indicated to determine if similar trends exist in this population. Traditionally, post‐cardiac arrest care in veterinary medicine has focused on hemodynamic stabilisation and neurologic recovery, often with limited emphasis on coagulation status. This case highlights a potential gap in post‐arrest management: when CPA occurs in association with interventional cardiac procedures such as balloon valvuloplasty, the likelihood of coagulation abnormalities including DIC or consumptive coagulopathy may be greater and clinically relevant. This report underscores the need to consider targeted coagulation testing in these scenarios to ensure early identification and management of potential complications, ultimately improving patient outcomes.

## Author Contributions

**Marissa A. Turner**: writing – original draft, writing – review and editing, resources, visualisation, conceptualisation, investigation. **Theresa C. Hallowell**: conceptualisation, writing – original draft, writing – review and editing, resources, supervision, visualisation, investigation.

## Ethics Statement

The authors confirm that the ethical policies of the journal, as noted on the journal's author guidelines page, have been adhered to. No ethical approval was required for this case report.

## Conflicts of Interest

The authors declare no conflicts of interest.

## Data Availability

The data that support the findings of this study are available from the corresponding author upon request.

## Endnotes

a Atenolol, Zydus Lifesciences Ltd., India.

b Element POC Blood Gas and Electrolyte analyzer, HESKA, Loveland, CO.

c Procainamide, Hospira, Lake Forest, IL.

d Maropitant, Zoetis Inc, Kalamazoo, MI.

e Butorphanol, Dechra, Overland Park, KS.

f Diazepam, CordenPharma S.P.A, Caponago, Italy.

g Propofol, SAGENT Pharmaceuticals, Schaumburg, IL.

h Sevoflurane USP, Piramal Critical Care Inc., Bethlehem, PA.

i Lidocaine, Covetrus, MWI, Boise, ID.

j Ketamine Hydrochloride, Vet One, Boise, ID.

k Cefazolin, Hikma Pharmaceuticals, Eatontown, NJ.

l Flumazenil, Hikma Pharmaceuticals, Eatontown, NJ.

m Naloxone HCL, Akorn Inc., Lake Forest, IL.

n Epinephrine VEDCO Inc. St. Joseph, MO.

o Atropine, MWI, Boise, ID.

p Lactated Ringer's solution, Baxter Corporation, Deerfield, IL.

q Dopamine, Hospira, Lake Forest, IL.

r Coag DX analyzer (Partial thromboplastin and prothrombin time), IDEXX, Westbrook, ME.

s Element ET5, Heska, Loveland, CO.

t Blood type quick test, Alvedia, Limonest, France.

u Packed red blood cells, in hospital blood bank.

v Fresh frozen plasma, Terumo Corporation, Tokyo, Japan.

w Tranexamic acid injectable, Auromedics Pharma, E. Windsor, NJ.

x Vitamin K1 injection, MWI, Boise, ID.

y Desmopressin, Wedgewood, San Jose, CA.

z Vasopressin, Par pharmaceutical, Chestnut Ridge, NY.

aa Hydrocortisone, Pharmacia & Upjohn Co. Division of Pfizer Inc., New York, NY.

ab Enrofloxacin 2.27% injection, Norbrook Laboratories Limited, Northern Ireland.

ac Metronidazole solution, API solutions, Daphne, AL.

ad Pantoprazole, Piramal Critical Care Inc., Bethlehem, PA.

ae Fentanyl, Hikma Pharmaceuticals, Berkeley Heights, NJ.

af Catalyst one chemistry analyzer, IDEXX, Westbrook, ME.

## References

[vms370445-bib-0001] Alkhouli, M. , C. J. Zack , M. Sarraf , et al. 2017. “Morbidity and Mortality Associated With Balloon Aortic Valvuloplasty: A National Perspective.” Circulation: Cardiovascular Interventions 10, no. 5: e004481. 10.1161/CIRCINTERVENTIONS.116.004481. 28495894

[vms370445-bib-0002] Bick, R. L. 2003. “Disseminated Intravascular Coagulation Current Concepts of Etiology, Pathophysiology, Diagnosis, and Treatment.” Hematology/Oncology Clinics of North America 17, no. 1: 149–176. 12627667 10.1016/s0889-8588(02)00102-8

[vms370445-bib-0003] Boon, J. A. 2011. Veterinary Echocardiography. 2nd ed. Wiley‐Blackwell.

[vms370445-bib-0004] Bussadori, C. , E. DeMadron , R. A. Santilli , and M. Borgarelli . 2001. “Balloon Valvuloplasty in 30 Dogs With Pulmonic Stenosis: Effect of Valve Morphology and Annular Size on Initial and 1‐Year Outcome.” Journal of Veterinary Internal Medicine 15, no. 6: 553–558. 11817060 10.1892/0891-6640(2001)015<0553:bvidwp>2.3.co;2

[vms370445-bib-0005] Duan, J. , H. Ge , W. Fan , et al. 2024. “Cardiac Arrest‐Associated Coagulopathy Could Predict 30‐Day Mortality: A Retrospective Study From Medical Information Mart for Intensive Care IV Database.” Clinical and Applied Thrombosis/Hemostasis 30: 10760296231221986. 10.1177/10760296231221986. 38196194 PMC10777779

[vms370445-bib-0006] Gando, S. , and T. Wada . 2019. “Disseminated Intravascular Coagulation in Cardiac Arrest and Resuscitation.” Journal of Thrombosis and Haemostasis 17, no. 8: 1205–1216. 31102491 10.1111/jth.14480

[vms370445-bib-0007] Goggs, R. , A. Mastrocco , and M. B. Brooks . 2018. “Retrospective Evaluation of 4 Methods for Outcome Prediction in Overt Disseminated Intravascular Coagulation in Dogs (2009‐2014): 804 Cases.” Journal of Veterinary Emergency and Critical Care 28, no. 6: 541–550. 10.1111/vec.12777. 30302935

[vms370445-bib-0008] Granger, K. L., Jr. , T. Paulos , M. K. Boss , L. Guieu , and S. Shropshire . 2024. “Case Report: Chronic Disseminated Intravascular Coagulopathy With Concurrent Paraneoplastic Secondary Hyperfibrinolysis in a Dog With Metastatic Nasal Adenocarcinoma.” Frontiers in Veterinary Science 11: 1375507. 10.3389/fvets.2024.1375507. 38840638 PMC11152169

[vms370445-bib-0009] Hardy, J. F. , P. de Moerloose , and C. M. Samama . 2006. “Massive Transfusion and Coagulopathy: Pathophysiology and Implications for Clinical Management.” Canadian Journal of Anaesthesia 53, no. Suppl 6: S40–S58. 10.1007/BF03022251. 16766790 PMC7103890

[vms370445-bib-0010] Honse, C. O. , F. B. Figueiredo , N. X. de Alencar , M. de Fátima Madeira , I. D. F. Gremião , and T. M. P. Schubach . 2013. “Disseminated Intravascular Coagulation in a Dog Naturally Infected by Leishmania (Leishmania) Chagasi From Rio de Janeiro—Brazil.” BMC Veterinary Research 9: 43. 10.1186/1746-6148-9-43. 23497531 PMC3599858

[vms370445-bib-0011] Johnson, M. S. , M. Martin , D. Edwards , A. French , and W. Henley . 2004. “Pulmonic Stenosis in Dogs: Balloon Dilation Improves Clinical Outcome.” Journal of Veterinary Internal Medicine 18: 656–662. 15515581 10.1892/0891-6640(2004)18<656:psidbd>2.0.co;2

[vms370445-bib-0012] Kapłon‐Cieślicka, A. , D. A. Kosior , M. Grabowski , A. Rdzanek , Z. Huczek , and G. Opolski . 2013. “Coronary Artery Dissection, Traumatic Liver and Spleen Injury After Cardiopulmonary Resuscitation—A Case Report and Review of the Literature.” Archives of Medical Science: AMS 9, no. 6: 1158–1161. 10.5114/aoms.2013.39235. 24482665 PMC3902713

[vms370445-bib-0013] Leblanc, N. L. , D. C. Silverstein , J. W. Ludders , et al. 2019. “Prevalence of Major Complications in Interventional Cardiology in 336 Dogs.” Journal of Veterinary Cardiology 24: 53–63. https://doi/10.1016/j.jvec.2018.11.004. 10.1016/j.jvc.2019.01.003 31174729

[vms370445-bib-0014] Levi, M. , and S. Sivapalaratnam . 2018. “Disseminated Intravascular Coagulation: An Update on Pathogenesis and Diagnosis.” Expert Review of Hematology 11, no. 8: 663–672. 10.1080/17474086.2018.1500173. 29999440

[vms370445-bib-0015] Levi, M. , C. H. Toh , J. Thachil , and H. G. Watson . 2009. “Guidelines for the Diagnosis and Management of Disseminated Intravascular Coagulation.” British Journal of Haematology 145: 24–33. 10.1111/j.1365-2141.2009.07600. 19222477

[vms370445-bib-0016] Majiyagbe, O. O. , A. M. Akinsete , T. A. Adeyemo , A. O. Salako , E. N. Ekure , and C. A. N. Okoromah . 2022. “Coagulation Abnormalities in Children With Uncorrected Congenital Heart Defects Seen at a Teaching Hospital in a Developing Country.” PLoS ONE 17, no. 7: e0263948. 10.1371/journal.pone.0263948. 35901057 PMC9333323

[vms370445-bib-0017] Padmanabhan, A. , M. R. Smith , V. Wurlod , J. C. Menk Pinto Lima , and F. Del Piero . 2024. “Acute Hepatic Rupture Causing Hemoperitoneum in a Dog With Anaphylaxis.” Veterinary Medicine and Science 10, no. 2: e1391. 10.1002/vms3.1391. 38403981 PMC10895156

[vms370445-bib-0018] Papageorgiou, C. , G. Jourdi , E. Adjambri , et al. 2018. “Disseminated Intravascular Coagulation: An Update on Pathogenesis, Diagnosis, and Therapeutic Strategies.” Clinical and Applied Thrombosis/Hemostasis 24, no. suppl 9: 8S–28S. 30296833 10.1177/1076029618806424 PMC6710154

[vms370445-bib-0019] Park, H. W. , S. H. Cho , K. H. Kim , and J. G. Cho . 2005. “Disseminated Intravascular Coagulation as a Complication of Radiofrequency Catheter Ablation of Atrial Fibrillation.” Journal of Cardiovascular Electrophysiology 16, no. 9: 1011–1013. 10.1111/j.1540-8167.2005.40800.x. 16174024

[vms370445-bib-0020] Philp, H. S. , K. S. Farrell , and R. H. L. Li . 2023. “Case Report: Disseminated Intravascular Coagulation in a Dog Following Treatment With Melarsomine for *Dirofilaria immitis* .” Frontiers in Veterinary Science 10: 1118798. 10.3389/fvets.2023.1118798. 36814463 PMC9939911

[vms370445-bib-0021] Quesada, J. , L. Londoño , G. J. Buckley , M. J. Dark , J. C. Colee , and L. L. Farina . 2021. “Retrospective Study of Gross and Histopathologic Lesions Associated With Closed Chest CPR in Dogs.” Journal of Small Animal Practice 62: 750–755. 10.1111/jsap.13349. 33987841

[vms370445-bib-0022] Ramos, R. V. , B. P. Monteiro‐Steagall , and P. V. Steagall . 2014. “Management and Complications of Anesthesia During Balloon Valvuloplasty for Pulmonic Stenosis in Dogs: 39 Cases (2000 to 2012).” Journal of Small Animal Practice 55, no. 4: 207–212. 24450439 10.1111/jsap.12182

[vms370445-bib-0023] Reich, O. 2004. “Long Term Results of Percutaneous Balloon Valvoplasty of Congenital Aortic Stenosis: Independent Predictors of Outcome.” Heart 90, no. 1: 70–76. 14676248 10.1136/heart.90.1.70 PMC1768014

[vms370445-bib-0024] Taylor, F. B., Jr. , C. H. Toh , W. K. Hoots , H. Wada , M. Levi , and Scientific Subcommittee on Disseminated Intravascular Coagulation (DIC) of the International Society on Thrombosis and Haemostasis (ISTH) . 2001. “Towards Definition, Clinical and Laboratory Criteria, and a Scoring System for Disseminated Intravascular Coagulation.” Thrombosis and Haemostasis 86, no. 5: 1327–1330. 11816725

[vms370445-bib-0025] Tucker, C. , A. Winner , R. Reeves , et al. 2022. “Resuscitation Patterns and Massive Transfusion for the Critical Bleeding Dog ‐ A Multicentric Retrospective Study of 69 Cases (2007‐2013).” Frontiers in Veterinary Science 8: 788226. 10.3389/fvets.2021.788226. 35071385 PMC8766795

[vms370445-bib-0026] Unger, P. , E. Stoupel , F. Delwiche , and G. Berkenboom . 1989. “Acute Disseminated Intravascular Coagulation After Percutaneous Balloon Aortic Valvuloplasty in a Cirrhotic Patient.” European Heart Journal 10, no. 3: 283–284. 2523308 10.1093/oxfordjournals.eurheartj.a059478

[vms370445-bib-0027] White, N. J. , B. S. Leong , J. Brueckner , et al. 2011. “Coagulopathy During Cardiac Arrest and Resuscitation in a Swine Model of Electrically Induced Ventricular Fibrillation.” Resuscitation 82, no. 7: 925–931. 10.1016/j.resuscitation.2011.02.034. 21482008 PMC3549665

[vms370445-bib-0028] Wiinberg, B. , A. L. Jensen , P. I. Johansson , et al. 2010. “Development of a Model Based Scoring System for Diagnosis of Canine Disseminated Intravascular Coagulation With Independent Assessment of Sensitivity and Specificity.” Veterinary Journal 185, no. 3: 292–298. 10.1016/j.tvjl.2009.06.003. 19586785

[vms370445-bib-0029] Wolberg, A. , M. Aleman , K. Leiderman , and K. Machlus . 2012. “Procoagulant Activity in Hemostasis and Thrombosis: Virchowʼs Triad Revisited.” Anesthesia and Analgesia 114, no. 2: 275–285. 10.17615/ntag-9429. 22104070 PMC3264782

[vms370445-bib-0030] Yang, W. , G. Hosgood , K. Luobikis , and A. Paul . 2018. “Agreement of Point‐of‐Care Prothrombin and Activated Partial Thromboplastin Time in Dogs With a Reference Laboratory.” Australian Veterinary Journal 96: 379–384. 10.1111/avj.12746. 30255579

